# Association between the longest job and oral health: Japan Gerontological Evaluation Study project cross-sectional study

**DOI:** 10.1186/1472-6831-14-130

**Published:** 2014-10-27

**Authors:** Tatsuo Yamamoto, Katsunori Kondo, Jun Aida, Shinya Fuchida, Yukio Hirata, for the JAGES group

**Affiliations:** Department of Dental Sociology, Graduate School of Dentistry, Kanagawa Dental University, Yokosuka, Japan; Center for Preventive Medical Science, Chiba University, Chiba, Japan; Center for Well-being and Society, Nihon Fukushi University, Nagoya, Japan; Department of International and Community Oral Health, Tohoku University Graduate School of Dentistry, Sendai, Japan

**Keywords:** Longest job, Oral health status, Oral health behavior, Older people, Cross-sectional study

## Abstract

**Background:**

Inequality in oral health is a major challenge. Oral diseases and their risk factors accumulate throughout life. The objective of this cross-sectional study was to examine the association of longest job with oral health status and oral health behavior among older Japanese.

**Methods:**

Subjects were a total of 23,191 (11,310 males and 11,881 females) community-dwelling individuals aged 65 or over, living independently and able to perform daily activities from 30 municipalities across Japan. The outcome variables were oral health status (number of teeth, use of denture or bridge and subjective oral health status) and oral health behavior (dental visit for treatment and use of interdental brush or dental floss). The longest job was used as an explanatory variable. Age, educational attainment, equivalent income, and densities of dentists and population in municipalities were used as covariates. Two-level (first level: individual, second level: municipality) multilevel Poisson regression analyses were performed for each sex.

**Results:**

Multilevel Poisson regression analyses showed that all variables of oral health status and oral health behavior were significantly associated with longest job after adjusting for all covariates except denture/bridge use and dental visit for females. People whose longest jobs were sales/service, skilled/labor, agriculture/forestry/fishery or others, or who had no occupation were more likely to have poor oral health status and oral health behavior compared to those whose longest jobs were professional/technical.

**Conclusions:**

The longest job may be one of the major determinants of oral health status and oral health behavior in Japanese older people.

## Background

As oral diseases and their risk factors accumulate throughout the course of life, meaning that inequality in oral health is a major challenge in an aging society [1]. Studies have shown socioeconomic inequalities in oral health status [2, 3] and oral health behavior [4] in older people.

Occupation has been used in epidemiological studies as a marker of socioeconomic status [5]. Occupational environment is important for health because people spend long hours at work during their lifetime. Therefore, the oral health of older people is affected by work time stress and health care policy in their occupational environment.

In Japan, some municipalities have oral health programs including periodontal examination for adult residents; however, participation rates (3.6% in 2002) are often not satisfactory [6]. Workplaces are suitable for oral health education and screening of periodontal disease because most of the target population would be involved. However, few workplaces have such systems [7].

Occupational classification is one marker of socioeconomic position and is associated with the health status of older people including the risk of chronic obstructive pulmonary disease [8], subclinical atherosclerosis [9] and chronic disabling pain [10]. Studies using employees in companies suggest gradients of oral health status in occupational classification [11, 12]. However, little is known about the association between occupational classification and oral health status and oral health behavior in older people. Particularly, no studies have been conducted to investigate the association between agriculture/forestry/fishery workers and oral health in older people.

It is useful for public health decision making and formulating a public health program to identify a target population from the viewpoint of occupational classification. The purpose of this study was to examine the association of the longest job (the job being done for the longest time) with oral health status and oral health behavior using cross-sectional data from community-dwelling older Japanese people. As a life-course study showed that low childhood socioeconomic status has a long-lasting negative influence on adult health including oral health [13], educational attainment and current economic status were taken into consideration in the analyses.

## Methods

### Study population

Data from a cross-sectional study, collected as part of the Japan Gerontological Evaluation Study (JAGES) Project, an on-going Japanese prospective cohort study, were used for this study. JAGES aims to conduct empirical studies from gerontological and social epidemiological perspectives. The sample was restricted to those who did not already have a physical or cognitive disability, defined by not receiving public long-term care insurance benefits, at baseline. From July 2010 to January 2012, a mail survey, exceptionally collected by visit in one prefecture, was conducted in a random sample of 169,215 community-dwelling individuals aged 65 years or over residing in 31 municipalities in 12 prefectures in Japan.

The questionnaire on oral health status and oral health behavior was sent to a random sample of 46,009 subjects, of which 27,732 (60.3%) responded. All subjects in one municipality were excluded because the questions used in the present study were not included in the questionnaire. After excluding 4,541 subjects who did not answer about the longest job held, a total of 23,191 subjects (11,310 males and 11,881 females) aged 65 or older in 30 municipalities were included in the present study.

### Outcome variables

Number of teeth, denture/bridge use and subjective oral health status were evaluated for oral health status, based on a self-administered questionnaire [14]. Respondents were asked to indicate the number of teeth as 20 or more, 10-19, 1-9 or 0. Denture/bridge use was ascertained by asking, “Do you use a denture or bridge?” with possible answers dichotomized into yes and no. Subjective oral health status was ascertained by asking, “How do you rate your oral health (teeth, gum and denture)?” with possible 4 grade answers dichotomized into good and poor.

Oral health behavior was evaluated based on dental visit for treatment and use of an interdental brush or a dental floss. Dental visit for treatment was ascertained by asking, “Did you visit a dentist for treatment (including adjustment of a denture) within 6 months?” with possible answers dichotomized into yes and no. Use of an interdental brush or a dental floss was ascertained by asking, “Do you use an interdental brush or dental floss?” with possible answers dichotomized into yes and no.

### Explanatory variable

Information on the longest job held was used as an explanatory variable and ascertained by asking, “What was the job that you had done for most of your working life?” with possible answers of professional/technical, administrative, clerical, sales/service, skilled/labor, and agriculture/forestry/fishery workers, others and no occupation.

### Covariates

Data on socio-demographics (sex, age, educational attainment, equivalent income) were obtained using a self-administered questionnaire and used as covariates. To adjust household income for household size, equivalent income was calculated by dividing the household income by the square root of the number of household members, and placed into one of seven categories (less than US$5,000, US$5,000-9,999, US$10,000-14,999, US$15,000-19,999, US$20,000-29,999, US$30,000-39,999, and US$4,000,000 or higher) (US$1 = 100 Japanese yen) [15].

Data on the number of dentists working in hospitals or clinics were obtained from the Survey of Physicians, Dentists and Pharmacists conducted by the Ministry of Health, Labour and Welfare, Japan in 2010. Data on population in 2010 and area of inhabitable land of each municipality (city, town or village) were obtained from the National Population Census Survey conducted by the Ministry of Internal Affairs and Communications, Japan. The number of dentists working in hospitals or clinics per 100,000 people (density of dentists) and population density were calculated for each municipality. The density of dentists was categorized into four groups (lowest, low middle, high middle, or highest) based on 25th, 50th, and 75th percentiles. Population density was categorized into four groups (metropolitan, urban, semi-urban, or rural-agricultural).

### Analysis

The percentage of respondents with poor oral health status and poor oral health behavior were calculated for each longest job in each sex. Two-level (first level: individual, second level: municipality) multilevel Poisson regression models with random intercepts and fixed slopes were used separately for males and females to calculate multilevel prevalence ratios (PRs) for the longest job after adjusting for age, educational attainment and equivalent income as individual-level variables and densities of dentists and population as municipality-level variables, with each variable of oral health status or oral health behavior as the outcome. In each model, subjects lacking a outcome variable were excluded from the analysis. However, covariates that included missing data were recorded by reassigning missing values to separate missing categories in order to maximize the number of subjects included in the statistical analysis and thereby maximize statistical power: data lacking information of educational attainment and equivalent income were included as “others or data missing” (the category of missing was combined with that of others) and “data missing” categories, respectively. Moreover, subjects with 20 or more teeth and those with 9 or less teeth were excluded in the models in which denture/bridge use and use of interdental brush/dental floss were used as outcome variables, respectively, because some subjects with 20 or more teeth and those with 9 or less teeth might not need to use denture/bridge and interdental brush/dental floss, respectively. All statistical analyses were performed using IBM SPSS Statistics 21 (International Business Machines Co., New York, NY, USA) and MLwiN 2.28 (Centre for Multilevel Modelling, University of Bristol, Bristol, UK).

### Ethical considerations

A detailed explanation of the objectives of the JAGES was sent by mail along with the self-administered questionnaire. People who were willing to participate in the study voluntarily completed and mailed back the questionnaire. The JAGES protocol and its informed consent procedure were reviewed and approved by the Ethics Committee on Research of Human Subjects at Nihon Fukushi University.

## Results

Distributions of the subjects in accordance with the longest job, age, educational attainment and equivalent income are shown in Table 1. In males, professional/technical workers (23.0%) followed by skilled/labor workers (20.9%) were most predominant; whereas, clerical workers (19.3%) followed by sales/service workers (18.9%) were most predominant among females. The percentage of female subjects having no occupation was 12.8%; however, that in males was 1.1%.

**Table 1:** **Distributions of the longest job, age, educational attainment and equivalent income in each sex**

		Male		Female
	N	%	N	%
Longest job				
Professional/technical	2,606	23.0	1,255	10.6
Administrative	1,305	11.5	142	1.2
Clerical	1,102	9.7	2,293	19.3
Sales/service	1,338	11.8	2,244	18.9
Skilled/labor	2,366	20.9	1,076	9.1
Agriculture/forestry/fishery	1,186	10.5	1,204	10.1
Others	1,280	11.3	2,152	18.1
No occupation	127	1.1	1,515	12.8
Age group (year old)				
65-69	3,148	27.8	3,249	27.3
70-74	3,407	30.1	3,496	29.4
75-79	2,562	22.7	2,596	21.9
80-84	1,498	13.2	1,582	13.3
85 or older	695	6.1	958	8.1
Educational attainment (year)				
Less than 6	279	2.5	519	4.4
6-9	4,739	41.9	5,462	46.0
10-12	3,625	32.1	4,041	34.0
13 or longer	2,483	22.0	1,590	13.4
Others or data missing	184	1.6	269	2.3
Equivalent income (US$)				
Less than 5,000	386	3.4	778	6.5
5,000-9,999	1,038	9.2	1,424	12.0
10,000-14,999	1,350	11.9	1,295	10.9
15,000-19,999	2,240	19.8	1,776	14.9
20,000-29,999	2,459	21.7	2,056	17.3
30,000-39,999	1,452	12.8	1,274	10.7
40,000 or higher	1,035	9.2	941	7.9
Data missing	1,350	11.9	2,337	19.7

Percentages of subjects with poor oral health status and poor oral health behavior are shown in Table 2. The percentage of males having no occupation and agriculture/forestry/fishery job was the highest in poor oral health status and poor oral health behavior; whereas, those with administrative and clerical job were the lowest. Percentages of females having sales/service, agriculture/forestry/fishery job and no occupation were the highest in poor oral health status and poor oral health behavior; whereas, those having professional/technical, administrative and clerical job were the lowest.

**Table 2:** **Total number of subjects for analyses and percentages of subjects with poor oral health status and poor oral health behavior according to the longest job in each sex**

Longest job in each sex	Poor oral health status						Poor oral health behavior			
	Having 19 or less teeth		Denture/bridge non-user in subjects with 19 or less teeth		Subjective poor oral health		No dental visit for treatment		Interdental brush/dental floss non-user in subjects with 10 or more teeth	
	N for analyses	%	N for analyses	%	N for analyses	%	N for analyses	%	N for analyses	%
Male										
Professional/technical	2,555	60.2	1,453	29.2	2,506	31.0	2,514	46.7	1,402	44.9
Administrative	1,281	57.5	701	25.8	1,256	24.5	1,269	40.7	755	43.8
Clerical	1,089	60.7	635	23.9	1,071	27.6	1,083	43.2	645	46.0
Sales/service	1,318	68.5	837	33.3	1,282	31.0	1,290	45.5	705	49.5
Skilled/labor	2,313	69.6	1,487	35.8	2,251	34.4	2,263	54.0	1,057	51.0
Agriculture/forestry/fishery	1,154	84.1	854	39.9	1,122	34.0	1,114	55.7	354	56.2
Others	1,238	71.1	787	40.2	1,200	33.2	1,201	51.9	536	53.7
No occupation	123	80.5	89	44.9	118	45.8	116	67.2	46	63.0
Total	11,071	66.8	6,843	33.1	10,806	31.3	10,850	48.7	5,500	48.4
Female										
Professional/technical	1,224	57.2	648	31.0	1,203	24.0	1,204	47.6	770	31.9
Administrative	132	68.9	82	29.3	132	22.0	132	50.8	57	28.1
Clerical	2,244	56.6	1,169	29.6	2,209	25.3	2,217	47.9	1,363	30.9
Sales/service	2,155	69.1	1,331	32.3	2,131	32.1	2,132	52.8	1,046	38.8
Skilled/labor	1,045	69.7	654	33.3	1,027	28.8	1,033	56.4	496	42.1
Agriculture/forestry/fishery	1,156	85.8	848	31.8	1,165	29.3	1,149	60.0	344	48.5
Others	2,084	74.5	1,366	34.8	2,042	29.8	2,036	55.0	865	44.3
No occupation	1,465	76.0	997	35.9	1,448	29.3	1,426	57.8	569	40.4
Total	11,505	69.0	7,095	32.7	11,357	28.4	11,329	53.3	5,510	37.7

Multilevel PRs of the longest job in each sex after adjusting for individual-level age, educational attainment and equivalent income, and municipality-level densities of dentists and population are shown in Table 3. Significantly high PRs for some kinds of longest jobs were observed in all variables of oral health status and all variables of oral health behavior except denture/bridge use and dental visit for treatment in females. Especially, agriculture/forestry/fishery workers showed significantly higher PRs in having 19 or less teeth (1.15, 95% confidence interval (CI): 1.06 - 1.26), denture/bridge non-users in subjects with 19 or less teeth (1.18, 1.01 - 1.36) and interdental brush/dental floss non-user in subjects with 10 or more teeth (1.21, 1.03 - 1.43) in males, and having 19 or less teeth (1.21, 1.09 - 1.34) and interdental brush/dental floss non-user in subjects with 10 or more teeth (1.25, 1.02 - 1.53) in females.

**Table 3:** **Fully adjusted multilevel prevalence ratios for the longest job in each sex**

	Poor oral health status						Poor oral health behavior			
Longest job in each sex	Having 19 or less teeth		Denture/bridge non-user in subjects with 19 or less teeth		Subjective poor oral health		No dental visit for treatment		Interdental brush/dental floss non-user in subjects with 10 or more teeth	
	PR	95% CI	PR	95% CI	PR	95% CI	PR	95% CI	PR	95% CI
Male										
Administrative	1.01	(0.92-1.10)	1.00	(0.84-1.20)	0.85	(0.74-0.97)*	0.91	(0.82-1.01)	1.02	(0.89-1.17)
Clerical	1.02	(0.93-1.12)	0.88	(0.73-1.06)	0.92	(0.80-1.05)	0.94	(0.84-1.05)	1.04	(0.91-1.20)
Sales/service	1.11	(1.02-1.21)*	1.08	(0.93-1.26)	0.98	(0.86-1.10)	0.97	(0.88-1.07)	1.08	(0.95-1.24)
Skilled/labor	1.07	(1.00-1.15)	1.11	(0.98-1.27)	1.08	(0.98-1.20)	1.13	(1.04-1.23)**	1.08	(0.96-1.22)
Agriculture/forestry/fishery	1.15	(1.06-1.26)**	1.18	(1.01-1.36)*	1.10	(0.96-1.25)	1.10	(1.00-1.22)	1.21	(1.03-1.43)*
Others	1.07	(0.98-1.16)	1.23	(1.06-1.42)**	1.05	(0.93-1.18)	1.08	(0.97-1.19)	1.15	(0.99-1.32)
No occupation	1.09	(0.89-1.34)	1.31	(0.94-1.82)	1.41	(1.06-1.87)*	1.30	(1.03-1.64)*	1.32	(0.91-1.93)
Female										
Administrative	1.16	(0.93-1.45)	1.00	(0.66-1.54)	0.94	(0.64-1.38)	1.07	(0.83-1.39)	0.87	(0.53-1.45)
Clerical	1.01	(0.92-1.11)	0.99	(0.83-1.18)	1.06	(0.92-1.23)	1.04	(0.94-1.16)	0.97	(0.83-1.14)
Sales/service	1.15	(1.05-1.26)**	0.99	(0.84-1.18)	1.29	(1.12-1.48)***	1.06	(0.96-1.17)	1.15	(0.97-1.35)
Skilled/labor	1.10	(0.99-1.22)	1.00	(0.82-1.22)	1.13	(0.96-1.33)	1.10	(0.98-1.24)	1.14	(0.94-1.38)
Agriculture/forestry/fishery	1.21	(1.09-1.34)***	0.95	(0.79-1.15)	1.16	(0.99-1.37)	1.04	(0.93-1.17)	1.25	(1.02-1.53)*
Others	1.15	(1.05-1.26)**	1.03	(0.87-1.22)	1.18	(1.02-1.36)*	1.06	(0.96-1.18)	1.23	(1.05-1.46)*
No occupation	1.13	(1.03-1.25)*	1.11	(0.93-1.33)	1.19	(1.02-1.39)*	1.10	(0.99-1.23)	1.14	(0.94-1.37)

PR, prevalence ratio; CI, confidence interval.

Reference: professional/technical.

Adjusted for individual-level age, educational attainment and equivalent income, and municipality-level densities of dentists and population.

*:*P* < 0.05, **:*P* < 0.01, ***:*P* < 0.001.

The municipality-level density of dentists was significantly associated with subjective oral health and dental visit for treatment in females in the fully adjusted models. Subjects in municipalities with a lower density of dentists were less likely to have subjective poor oral health and visit dentists. Municipality-level population density was significantly associated with the number of teeth and dental visit for treatment in females. Subjects in municipalities with lower population density were less likely to have 20 or more teeth and visit dentists.

## Discussion

The results of the present study showed that the longest job held was significantly associated with oral health status and oral health behavior in older Japanese people even after adjusting for individual-level age, educational attainment (childhood socioeconomic status) and equivalent income (current socioeconomic status) and municipality-level differences in densities of dentists and population (environment related to access to dental care). Especially, older people whose longest jobs were sales/service, skilled/labor, agriculture/forestry/fishery or others, or who had no occupation were more likely to have poor oral health status and poor oral health behavior compared to those whose longest jobs were professional/technical.

The results of present study agree with those of previous studies [11, 12, 16]. Studies in Japanese male workers aged 20-69 years showed that professional and office workers had better oral health including periodontal status and number of teeth than salespersons and service occupations [11, 12]. A study from Denmark among 75-year-olds, in which the longest job held was categorized into unskilled workers, skilled workers, low managerials, high managerials and others, showed that unskilled workers had significantly higher odds ratios for having no or few teeth than high managerials and others after adjusting for income and education [16]. In addition to support the results of the previous studies, the present study added new findings including that agriculture/forestry/fishery job was one of the occupational classes associated with poor oral health status and poor oral health behavior.

Sex differences were observed in the association between the longest job held and some variables of oral health status and oral health behavior. However, no statistically significant differences in the longest job held were observed in denture/bridge use in females after adjusting for individual-level age, education and income, and municipality-level densities of dentists and population. A significant association was observed between low income and not using denture/bridge (data not shown). These results suggest that the difference in denture/bridge use was ascribed to differences in the present economic status but not the longest job in females. Denture/bridge treatment is covered by insurance in Japan; however, 10-30% of the treatment fee is out-of-pocket. Therefore, the financial aspect of dental treatment should be considered in order to reduce oral health inequalities especially in females.

There are several possible pathways between the longest job held and oral health status and oral health behavior. One possibility is that temporal accessibility to dental health care varies according to occupational classification. For example, professional workers could manage their schedule by themselves and could easily gain access to dental health care. However, sales/service and skilled/labor workers may have a lesser degree of time flexibility than professional workers. In addition to the time flexibility according to the longest job, present working situation according to the longest job may affect denture/bridge use and dental visit. The percentage of subjects who still work was highest in agriculture/forestry/fishery workers (47% for males, 36% for females). Because agriculture/forestry/fishery workers still work, they might have less chance to visit dentists and receive denture treatment.

A second possibility is that spatial accessibility differs among the job type. Agriculture/forestry/fishery workers live in rural areas. On the other hand, professional/technical workers live in urban areas. The density of dental clinics is higher in urban areas than rural areas in Japan, especially in the period when the study subjects were engaged in their longest job [17]. A study showed that the density of dentists was associated with having a regular dentist in Japan even where universal healthcare insurance covered dental care [18]. Fully adjusted models in the present study showed that densities of dentists and population were significantly associated with some variables of oral health status and oral health behavior. As even after adjusting for densities of dentists and population, the longest job held was associated with oral health status, the longest job itself might be a significant determinant of oral health status.

The third possibility is that the perceived need of dental health varies according to job type. For example, professional/technical, administrative or clerical workers are more likely to pay attention to their appearance and speech, which are affected by the front teeth, than agriculture/forestry/fishery workers and people without an occupation. The fourth possibility is that the chance to participate in an oral health program and to obtain information of oral health varies among the longest jobs. Oral health programs including dental check-up are not mandated by law in Japan. Large companies have their own oral health program; however, small companies and small municipalities where agriculture/forestry/fishery workers live do not [19].

The strengths of the present study include large sample size, population-based sampling, and control for potential confounding factors. However, the present study also has a number of limitations. First, measurement of oral health status was based on self-report, not based on clinical examination. However, the validity and reliability of the self-reported number of teeth and denture-bridge use has been established by multiple studies and widely used in epidemiological surveys [20]. We have confirmed the validity of our questionnaire for oral health status [14]. Second, data on the longest job held were also obtained using a self-administered questionnaire. However, the validity of the measure was reported [21]. Third, duration spent in the longest job was unknown. For example, housewives who worked in a technical field for a year before marriage were counted as “technical workers” rather than no occupation even if the non-working period was longer than the working period. A further study considering the duration is needed to confirm the results of the present study. Fourth, the characteristics of the analyzed population might not represent that of the target population. Because demographic data on the target population were not obtained, we could not evaluate representativeness of the analyzed population. Our previous study, which was done in 2003 in one municipality using the same survey method (response rate: 55.5%), showed that people under the age of 80 and with middle to high levels of household income were more likely to respond to a questionnaire survey [22].

## Conclusions

The longest job held was significantly associated with oral health status and oral health behavior in older Japanese people even after adjusting for individual-level age, educational attainment and equivalent income, and municipality-level densities of dentists and population. Especially, older people whose longest jobs were sales/service, skilled/labor, agriculture/forestry/fishery or others, or who had no occupation were more likely to have poor oral health status and poor oral health behavior compared to those whose longest jobs were professional/technical. These results suggest that intervention in people whose jobs were sales/service, skilled/labor, agriculture/forestry/fishery or others, or who had no occupation may help reduce inequality in oral health due to job classification.

## Abbreviations

JAGES: Japan Gerontological Evaluation Study
PR: Prevalence ratio
CI: Confidence interval.
